# Sustainable Cellulose Nanofibers-Mediated Synthesis of Uniform Spinel Zn-Ferrites Nanocorals for High Performances in Supercapacitors

**DOI:** 10.3390/ijms24119169

**Published:** 2023-05-24

**Authors:** Lucas T. Teixeira, Scarllet L. S. de Lima, Taissa F. Rosado, Liying Liu, Hector A. Vitorino, Clenilton C. dos Santos, Jhonatam P. Mendonça, Marco A. S. Garcia, Rogério N. C. Siqueira, Anderson G. M. da Silva

**Affiliations:** 1 Departamento de Engenharia Química e de Materiais—DEQM, Pontifícia Universidade Católica do Rio de Janeiro, Rio de Janeiro 22451-040, RJ, Brazil; 2 Centro Brasileiro de Pesquisas Físicas, Rio de Janeiro 22290-180, RJ, Brazil; 3 Centro de Investigación en Biodiversidad para la Salud, Universidad Privada Norbert Wiener, Lima 15046, Peru; 4 Departamento de Física, Centro de Ciências Exatas e Tecnologia, Universidade Federal do Maranhão, São Luís 65080-805, MA, Brazil; 5 Departamento de Química, Centro de Ciências Exatas e Tecnologia, Universidade Federal do Maranhão, São Luís 65080-805, MA, Brazil

**Keywords:** supercapacitors, Zn-ferrites, controlled nanomaterials, nanocorals

## Abstract

Spinel ferrites are versatile, low-cost, and abundant metal oxides with remarkable electronic and magnetic properties, which find several applications. Among them, they have been considered part of the next generation of electrochemical energy storage materials due to their variable oxidation states, low environmental toxicity, and possible synthesis through simple green chemical processing. However, most traditional procedures lead to the formation of poorly controlled materials (in terms of size, shape, composition, and/or crystalline structure). Thus, we report herein a cellulose nanofibers-mediated green procedure to prepare controlled highly porous nanocorals comprised of spinel Zn-ferrites. Then, they presented remarkable applications as electrodes in supercapacitors, which were thoroughly and critically discussed. The spinel Zn-ferrites nanocorals supercapacitor showed a much higher maximum specific capacitance (2031.81 F g^−1^ at a current density of 1 A g^−1^) than Fe_2_O_3_ and ZnO counterparts prepared by a similar approach (189.74 and 24.39 F g^−1^ at a current density of 1 A g^−1^). Its cyclic stability was also scrutinized via galvanostatic charging/discharging and electrochemical impedance spectroscopy, indicating excellent long-term stability. In addition, we manufactured an asymmetric supercapacitor device, which offered a high energy density value of 18.1 Wh kg^−1^ at a power density of 2609.2 W kg^−1^ (at 1 A g^−1^ in 2.0 mol L^−1^ KOH electrolyte). Based on our findings, we believe that higher performances observed for spinel Zn-ferrites nanocorals could be explained by their unique crystal structure and electronic configuration based on crystal field stabilization energy, which provides an electrostatic repulsion between the d electrons and the p orbitals of the surrounding oxygen anions, creating a level of energy that determines their final supercapacitance then evidenced, which is a very interesting property that could be explored for the production of clean energy storage devices.

## 1. Introduction

Although the depletion of fossil fuel reserves is worrisome due to our natural resources’ energy dependence, the climate change caused by their increased utilization has created a critical necessity for developing efficient, renewable, and clean energy production and storage processes [1,2]. However, alternative technologies (e.g., solar and wind energies) are not competitive with traditional methods due to their high fluctuations [3]. Thus, to help to reduce such issues, supercapacitors (SCs) are important since they can be used as energy storage devices in renewable generation plants to restart power systems or provide energy until the energy source stabilizes, i.e., they can charge and discharge systems preventing current spikes, and therefore, provide almost endless charging cycles with much higher charging speeds [4]. In this case, several classes of nanomaterials have attracted special interest for application as SCs, including carbon [5], metal oxides [6], metal-organic frameworks (MOFs) [7], zeolites [8], spinel ferrites [9], and composites [10].

Among them, the spinel ferrites—MFe_2_O_4_ (M = Co, Cu, Ni, among others)—and their composites have been implemented as potential materials for SCs applications due to their low-cost production features [11,12]. For such purposes, Zn-based ferrites are widely considered as electrode materials for storage issues due to their redox properties, chemical stability, non-toxicity, and high storage capacity [13,14,15,16]. Additionally, they present singular electronic and magnetic properties, flexible synthesis routes, high abundance, and biocompatibility. In addition, they offer high versatility, which is well-discussed in several previous reviews on their various applications, from biomedical, sensors, water treatment, and electrochemical utilization to catalytic applications [17,18,19,20,21]. In particular, SCs based on spinel ferrites are expected to be especially interesting in this regard since the ferrite elements (Fe and O) are the first and fourth most abundant elements of the earth’s crust, respectively, with most of this crustal iron being found a with a spinel lattice structure with co-existence of both M^2+^ and Fe^3+^ ions in their spinel lattice [19].

Interestingly, many reports have described synthesis procedures based on chemical co-precipitation [22,23], microemulsion [24], solid-state reactions [25,26], thermal decomposition [27,28], and hydrothermal processes [29,30]. However, most previously reported procedures led to poorly controlled materials. In this case, it is well-established that fine control over these aspects is crucial to achieving higher performances and stability as the energy storage capacity is strongly dependent upon their particle size, shape (crystal facets), chemical composition, and crystal structure [31]. On the other hand, SCs are highly structure sensitive as it involves both the charge transfer species and the interface process [32]; thus, nanostructure engineering can be seen as an effective method to tune their final electrochemical performances [33]. Among the various morphologies, three-dimensional (3D) porous nanocorals have drawn extensive research interest due to porous mass-transfer channel, small particle size, and stable structure, which endow both charge transfer and interface processes with abundant active sites and high specific surface area [34,35,36,37].

Additionally, alternative green synthesis protocols have attracted relevant attention as new regulators for the design of controlled nanomaterials. Among them, biomass-based methods, such as those based on applying cellulose, chitosan, and starch, are advantageous due to their abundance and availability as polymers. Such biopolymers can efficiently act as regulators during nanostructures’ synthesis [38]. In this context, polysaccharides nanofibers, such as nanocellulose, have attracted considerable attention from the scientific community, which can be explained by both the high surface area that can be achieved (typically around 153–284 m^2^/g) [38], and the presence of hydroxyl or carboxylate groups when oxidized under the presence of TEMPO [39,40], building interesting sites for controlled nanostructures growth [39]. Additionally, their interaction with the nanostructures obtained can be weak enough (coulombic interaction), making it easy to separate the nanostructure from nanofibers through simple physical processes, such as solvent extraction [39].

Some authors have recently proposed using nanocellulose fibers as a template for synthesizing oxide nanocrystals through prior metals adsorption and subsequent heat treatment under an oxidant atmosphere [41]. In this processing route, the nanofibers react with oxygen and the metals are absorbed to the functional groups. Since metal adsorption is a prior step to nanostructure formation, it should occur with considerable driving force and kinetics. Both aspects are observed when the nanofibers are functionalized through the substitution of hydroxyl units by carboxylate groups (TCNF), as demonstrated by the high potential for Co^2+^ adsorption from aqueous solutions [40,42].

In this study, it is reported a controlled and eco-friendly synthesis of zinc and iron-containing oxide nanocorals, including a Zn-ferrite spinel, mediated by TEMPO-oxidized cellulose nanofibers (TCNF), displaying high surface area and multiple hydroxyl carboxylate groups, for suitable adsorption of Zn^2+^ and Fe^3+^ cations from aqueous solutions, followed by a controlled thermal oxidation under synthetic air atmosphere, to produce the desired metallic oxide nanomaterials, and concomitantly chemically react with nanocellulose, which is converted to gaseous molecules. In all cases, particles with uniform size, composition, and crystal structure have been observed. Additionally, the use of TCNF offered benefits for the easy manufacture of low-cost materials that resulted in enhanced electrode performance, which can be a promising alternative for supercapacitors engineering. Our studies provided a facile method to prepare well-defined nanostructures that do not need expensive reagents or sometimes prohibitive laboratory needs. Moreover, results demonstrated that regarding the spinel-containing sample, a supercapacitance behavior was observed (2031.81 F g^−1^ at a charge/discharge current density of 1 A g^−1^ in a 2.0 mol L^−1^ KOH electrolyte), which was absent in both pure Fe_2_O_3_ and ZnO samples produced with the same synthesis protocol. For these samples, much lower capacitance values were measured, which have proven to be equal to 184 F g^−1^ for Fe_2_O_3_ and 24.39 F g^−1^ for ZnO. We also manufactured an asymmetric supercapacitor device, which offered a high energy density value of 18.1 Wh kg^−1^ at a power density of 2609.2 W kg^−1^ (at 1 A g^−1^ in 2.0 mol L^−1^ KOH electrolyte).

## 2. Results and Discussion

**Figure 1:**
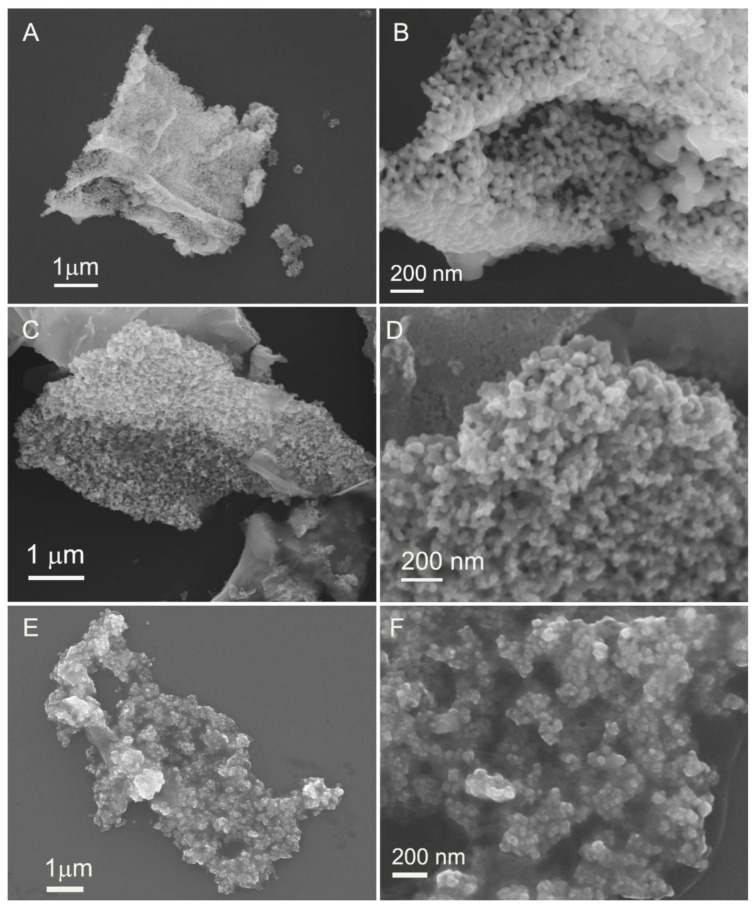
Low-magnification and corresponding high-resolution SEM images of (**A**,**B**) Fe_2_O_3_; (**C**,**D**) ZnO; and (**E**,**F**) Zn-ferrite nanocorals.

**Figure 2:**
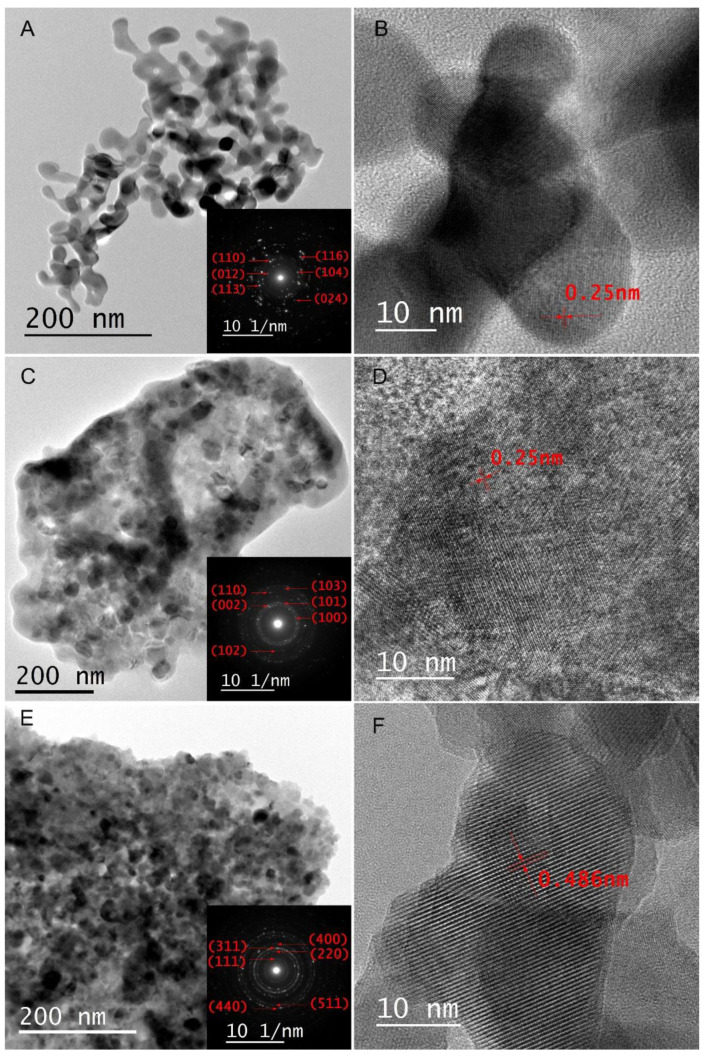
Low-magnification TEM and corresponding HRTEM images of (**A**,**B**) Fe_2_O_3_; (**C**,**D**) ZnO; and (**E**,**F**) Zn-ferrite. Inset shows the SAED pattern of each sample. The patterns were indexed based on the standard powder X-ray diffraction pattern with the PDF card number: 00-011-1053 for Fe_2_O_3_, 00-011-1136 for ZnO, and 00-001-1108 for ZnFe_2_O_4_.

**Figure 3:**
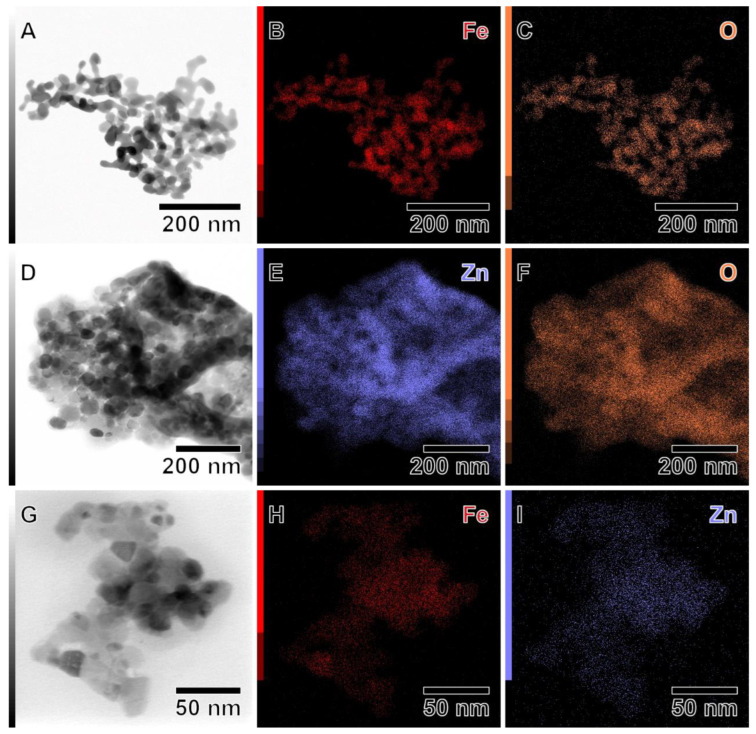
STEM images and EDS elemental mapping for (**A**–**C**) Fe_2_O_3_ nanocorals; (**D**–**F**) ZnO nanocorals; and (**G**–**I**) Zn-ferrite nanocorals.

The Zn-ferrite spinel (Fe_2_ZnO_4_), ZnO, and Fe_2_O_3_ nanocorals were prepared by an eco-friendly procedure using inexpensive reactants (as described in the experimental section). The syntheses were mediated by the use of TCNF (displaying high surface area and multiple hydroxyl/carboxyl groups units along the polymeric chains) as templates for suitable adsorption of Zn^2+^ and Fe^3+^ cations from an aqueous solution, followed by thermal treatment at 500 °C in the air (muffle furnace) for 2 h. Low-magnification SEM images indicate a porous morphology of all the samples. The high-resolution SEM images show a multiply linked structure formed by uniform and well-defined nanoparticles with sizes in the range of 20–30 nm, as shown in Figure 1A–F. Furthermore, the porous morphology and visible boundaries of the material suggest a particle attachment growth mechanism, which can be confirmed by the HRTEM images (Figure 2A–F). The particles were formed, then attached to each other through a self-assembly process. As a result, nanocoral-shaped structures were obtained for all samples. The HRTEM images of individual Zn-ferrite, ZnO, and Fe_2_O_3_ nanoparticles composing the nanocorals showed that all samples are polycrystalline. This observation was confirmed by the selected area electron diffraction (SAED) patterns, as shown in the insets of Figure 2, showing bright dots and circles. The SAED patterns were indexed based on each sample’s standard X-ray diffraction patterns. The distance between the atomic planes in Figure 2B was measured as 0.25 nm, corresponding to the value of the (110) plane of Fe_2_O_3_. The value shown in Figure 2D follows the (101) plane of ZnO, and in Figure 2F, the measured 0.486 nm agrees with the distance between (111) planes of ZnFe_2_O_4_. STEM-EDS elemental mapping results (Figure 3A–I) show the uniform distribution of the elements that form the materials (Zn, Fe, and O), although the samples show nanocoral structure. The results agree with the supposed particle-attachment growth mechanism. In addition, the HRTEM study confirmed the size observed in the SEM images.

The textural properties of spinel Zn-ferrites, ZnO, and Fe_2_O_3_ nanocorals researched by nitrogen physisorption are depicted in Table S1. The specific surface area and BJH pore diameter of spinel Zn-ferrites, Fe_2_O_3,_ and ZnO nanocorals corresponded to 33, 31, and 20 m^2^ g^−1^; and 24, 27, and 17 nm, respectively. The adsorption isotherms of all the samples (Figure S1) could be assigned to type IV (IUPAC classification), which is typical of mesoporous materials [43]. The shape of the hysteresis loop perfectly matches the H3 type with well-defined loops that did not level off at relative pressures close to the saturation vapor pressure. This shape is usually noticed due to differences in the behavior of the material in the adsorption and desorption processes. Such hysteresis loops are generally found on agglomerated solids, which follow the described self-assembly process observed in the HRTEM, with visible boundaries that suggest a particle attachment growth mechanism.

Figure S2 reveals the crystalline phase and structure of the Zn-ferrite, zincite (ZnO), and (hematite) Fe_2_O_3_ nanocorals according to XRD patterns. As shown in Figure S2, all Zn-ferrite diffraction peaks can be well assigned to cubic spinel phase franklinite (Fe_2_ZnO_4_) of cubic structure with the most prominent peaks located at Bragg angles equal to 2θ = 18.1°, 30.0°, 35.3°, 42.9°, 53.2°, 56.7°, 62.3°, 73.7°, and 89.3°, respectively, related to the hkl coordinates (111), (022), (113), (004), (224), (115), (044), (335), and (553) (JCPDS No. 96–901–2442), respectively. The XRD results also indicated the presence of lower intense peaks related to a residual amount of a rhombohedral hematite structure, which is located at Bragg angles equal to 33.2°, 48.8°, 54.1°, and 64.0°, respectively, corresponding to lattice indexes equal to (111), (222), (333), and (444) (JCPDS), respectively. Regarding the synthesized ZnO nanocorals (Figure S2), the obtained XRD pattern presented the most prominent peaks for Bragg angles located at 31.8°, 34.4°, 36.3°, 47.6°, 56.6°, 62.8°, and 68.0°, respectively, which can be ascribed to the lattice indexes (100), (002), (101), (201), (011), (301), and (211), lattice indices, respectively. The data is consistent with the sole presence of zincite, a hexagonal crystalline phase (JCPDS n^o^ 00-036-1451). Finally, the Fe_2_O_3_ nanocorals (Figure S2) showed peaks at 2θ = 24.2°, 33.2°, 35.6°, 40.9°, 50.0°, 54.1°, and 64.0, which are attributed to the (210), (401), (011), (311), (420), (611), and (003) lattice indices, respectively, which are coherent with the hematite phase JCPDS No. 39-1346.

The results from peak indexing analysis were also confirmed through Rietveld refinement of the measured diffraction patterns (Figures S3–S5). A good refinement was achieved for the Zn-ferrite and zincite samples, with GOF values equal to 1.21 and 2.15, respectively. The slight deviations explain the higher GOF for the zincite sample between experimental data and modeling for the two most prominent peaks, located around 31.8 and 36.2, respectively. These small deviations should most probably be assigned to some preferential orientation effect. Likewise, it can be observed that in the case of the Zn-ferrite sample, small amounts of iron sulfates have been detected, which can be explained by the fact that the precursor used was a sulfate in the case of iron.

Moreover, the obtained lattice parameters agree with the literature for both samples (see Table S2), especially for the Zn-ferrite spinel phase. In the case of the hematite sample, a much higher GOF was found (3.02), and a corresponding much more significant deviation for the c parameter was found (see Table S2). A very strong preferential orientation tendency can explain the difficulty during refinement. It is clear from Figure S3 that all peaks, which were not fitted to the model, are associated with Bragg angles characteristic of the α-hematite phase of rhombohedral structure, and therefore, should not be related to some impurity or some other crystal structure for the same material. This stronger preferential orientation of the sample could be related to its unique nanostructure, of “nanocoral type”, as depicted by TEM and SEM analysis, exhibiting much flatter surfaces. Finally, the mean crystallite sizes were in all cases of a similar order of magnitude, varying from 26.3 nm for zincite to 52.8 nm for the spinel sample. These values suggest the presence of crystals with sizes far in the nanoscale, also confirmed by TEM analysis (Figure 2).

**Figure 4:**
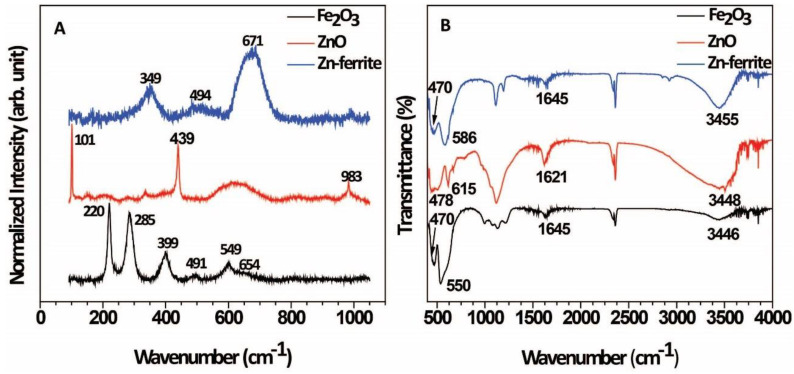
(**A**) Raman and (**B**) FTIR spectra for Zn-ferrite, ZnO, and Fe_2_O_3_ nanocorals.

Figure 4A presents the Raman spectrum of the Zn-ferrite nanocorals. In the range of 100–1000 cm^−1^, the Raman spectrum shows signals from a cubic Zn-ferrite structure (space group Fd3m), displaying five active Raman modes (A_1g_ + E_g_ + 3_F2g_) [44]. These five Raman modes are observable at 220, 285, 399, 491, 549, and 654 cm^−1^, respectively. The motion of oxygen in tetrahedral A_O4_ groups occurred at the modes above 600 cm^−1^; hence, the mode at 654 cm^−1^ can be assigned to the A_1g_ symmetry, and the other low-frequency modes correspond to both Eg (mode at 285 cm^−1^) and F_2g_ (modes at 220, 399, and 491 cm^−1^), which represent the characteristics of the octahedral sites (BO_6_). The Raman spectrum for ZnO nanocorals shows two intense peaks in the ZnO located at 101 and 439 cm^−1^, resulting from two degenerate E_2_ phonon modes of the Zn and O sublattice vibration; specifically, E_2_ low and E_2_ high, respectively [41]. The peak observed at 983 cm^−1^ is related to a second-order mode, characteristic of ZnO nanostructures. Finally, the Raman spectrum for Fe_2_O_3_ nanocorals (Figure 4A) presents the D^6^_3d_ crystal space group, corresponding to several A_1g_ and E_g_ phonon modes [42]. The peaks at 220 and 491 cm^−1^ were assigned to the A_1g_ phonon modes, while the peaks at 285, 399, 491, and 654 cm^−1^ are related to E_g_ phonon modes, indicating that nanostructure is composed of α-Fe_2_O_3_. No other iron oxide, such as magnetite or maghemite, was detected, which agrees with XRD results.

The Zn-ferrites, and ZnO and Fe_2_O_3_ nanocorals were also researched by FTIR (Figure 4B). The FTIR spectrum of spinel Zn-ferrites nanocorals in the range 400–4000 cm^−1^ presents two intrinsic bands, corresponding to υ_1_ = 586 cm^−1^, related to the intrinsic stretching vibrations of metal at the tetrahedral site (M_tetra_-O), while the band centered at υ_2_ = 470 cm^−1^ could be assigned to the octahedral metal stretching vibration (M_octa_−O). On the other hand, The FTIR spectra of ZnO nanocorals (Figure 4B) present the characteristic stretching mode of Zn-O. In this case, the IR spectrum of ZnO manifests the characteristic absorption band at 478 and 615 cm^−1^ due to the stretching vibration modes of Zn-O [45]. Finally, the FTIR data for Fe_2_O_3_ nanocorals indicated the presence of bands at 470 and 550 cm^−1^, which can be attributed to the Fe–O stretching vibration modes in α-Fe_2_O_3_ [46]. Moreover, the bands centered at 3455 cm^−1^ and less intense peak at 1612 cm^−1^ observed for all samples are typically related to the stretching and bending vibrations of H-O-H at the nanostructure surface. The intense peak found for all samples, approximately at 2360 cm^−1^_,_ can be assigned to absorb CO_2_ molecules present in the equipment atmosphere.

**Figure 5:**
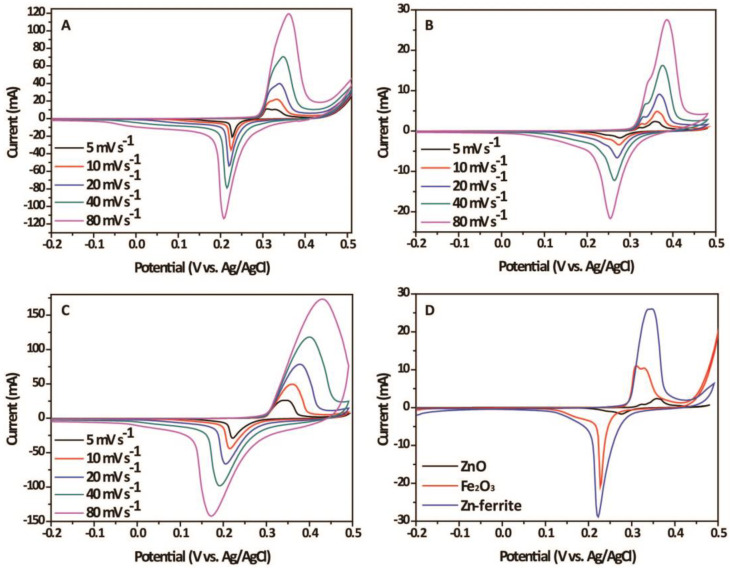
CV curves for (**A**) Fe_2_O_3_, (**B**) ZnO, and (**C**) Zn-ferrite nanocorals at different scan rates. (**D**) Comparison of CV curves of the three samples at 5 mV s^−1^. All the experiments were recorded at 2.0 M KOH solution.

In the next step, we focused on applying the prepared nanocorals for storage applications. The cyclic voltammetry (CV) curves at different scan rates (5–80 mVs^−1^) for the Fe_2_O_3_, ZnO, and Zn-ferrite nanocorals samples shown in Figure 5. The profiles exhibited a pseudocapacitive-like nature for all the cases, although the materials did not present a characteristic rectangular shape [12,47]. Additionally, as expected, reduction peaks shift to lower potentials with the scan rate increase, while the contrary is observed for the oxidation peaks, i.e., the shift is for higher potentials. Slight differences in the redox peaks are observed due to the Fe_2_O_3_ and ZnO different structures (Figure 5A,B). However, the synergy raised from the combination of both metals for the preparation of the Zn-ferrite can be noted by its CV profiles (Figure 5C). One can notice that the oxidation peaks shift to even higher voltages with the scan rate increase when compared to the monometallic counterparts; additionally, the reduction peak moved toward lower potentials.

Specifically, regarding the Zn-ferrite nanocorals (Figure 5C), there are two redox peaks at low scan rates, equivalent to a reversible redox reaction, in which one redox peaks pair for the ZnFeO electrode are observed, which could be possibly caused by the Faradaic process of Zn/Zn^2+^ and Fe/Fe^2+^/Fe^3+^. The material presents an anodic peak at 0.42 V and the corresponding cathodic peak potential at 0.16 V, which probably is responsible for the reversible electrochemical reactions from ZnFeO to ZnOOH and FeOOH species, as follows [48]:

ZnFeO + H_2_O + OH^−^ → ZnOOH + FeOOH + e^−^

ZnOOH + OH^−^ → ZnO_2_ + H_2_O + e^−^

FeOOH + OH^−^ → ZnO_2_ + H_2_O + e^−^

**Figure 6:**
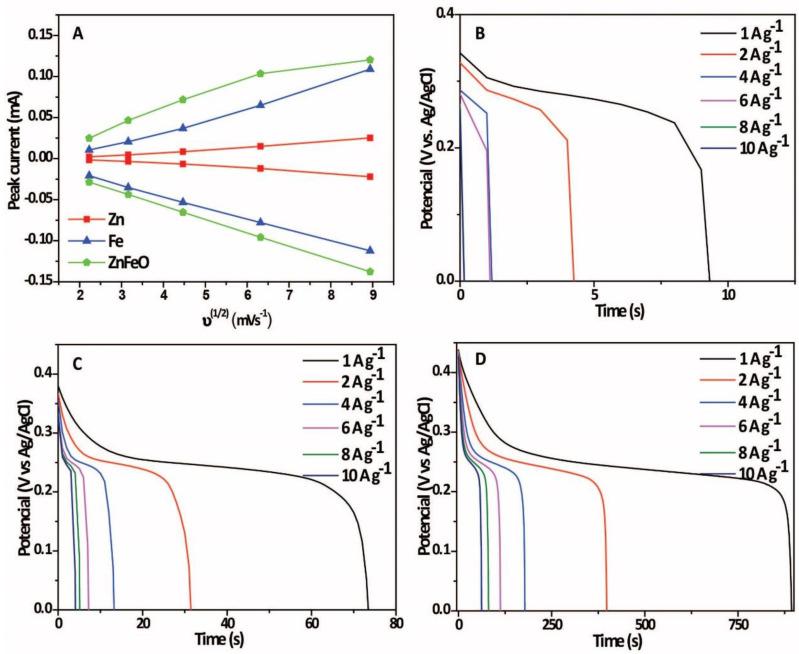
(**A**) Peak current (anodic: ascendent lines; cathodic: descendent lines) as a function of scan rate, (**B**) GCD profiles for ZnO, (**C**) Fe_2_O_3_, and (**D**) Zn-ferrite nanocorals electrodes at 1, 2, 4, 6, 8, and 10 A g^−1^.

Figure 5D shows a comparison among the samples related to their obtained CV curve at 5 mVs^−1^. Although different redox events occur comparing ZnO and Fe_2_O_3_ nanocorals, it should be noted that the signal of the ZnO nanocorals is lower. Additionally, comparing both materials with the Zn-ferrite nanocorals, the latter shows remarkable current gain, which was expected to be reflected in their performances. As important information regarding the prepared nanosystems, Figure 6A shows the association between oxidation and reduction peak currents and the square root of scan rate (v^1/2^), which offers a quasilinear relationship, indicating that the electrode reactions are diffusion-controlled with OH^−^ diffusion from the electrolyte to the electrodes’ surface involved [49].

It is important to note that previous reports showed that spinel crystal structures of ferrites could be classified as (i) “normal spinel” and (ii) “inverse spinel”, which occur more commonly. More specifically, Ni-, Co-, Mg-, and Cu-ferrites are listed as inverse spinels. In this case, Ni, Co, Mg, and Cu elements do not entirely take over the octahedral sites but are distributed between tetrahedral and octahedral sites. Generally, two hypotheses can be proposed to justify the ferrites’ preference to adopt inverse or normal spinel: the ion-size effect and the crystal field stabilization energy (CFSE). In the first rationale, the smaller cations occupy the smaller interstitial sites (tetrahedral sites), and the larger ions occupy the larger sites (octahedral sites). Since the M^2+^ ions are generally larger than the Fe^3+^ ion (the Fe^3+^ ion with a larger charge pulls the outer orbital and therefore is smaller than M^2+^), the M^2+^ ions occupy the octahedral sites. This logic justifies the behavior of most ferrites, being inverse spinels.

On the other hand, the normal spinel ferrites (Mn- and Zn-ferrites) may be justified by electronic configuration based on CFSE. The CFSE originates from the spatial arrangement of cation’s d orbitals and their environment. An electrostatic repulsion between the d electrons and the p orbitals of the surrounding oxygen anions creates a level of energy that determines the system’s stability. We believe this unique crystal structure and the expected electrostatic repulsion between the d electrons and the p orbitals at the spinel crystal structure could strongly affect their activities, leading to superior SCs performances relative to their monometallic counterparts. It is important to note that during Rietveld’s analysis of the Zn-ferrite sample, the structure considered for the spinel phase was of normal type and stoichiometric, corroborating the CFSE arguments cited before.

In this sense, to evaluate whether the described characteristic will affect the performance of the materials, GCD curves for the electrodes are displayed in Figure 6B–D, which were measured at different discharge current densities of 1, 2, 4, 6, 8, and 10 A g^−1^. All the materials present only one plateau, consistent with the CV curves. The Zn-ferrite nanocorals showed a much higher maximum specific capacitance (2031.81 F g^−1^ at a current density of 1 A g^−1^) than Fe_3_O_4_ and ZnO counterparts (189.74 and 24.39 F g^−1^ at a current density of 1 A g^−1^, respectively). The following specific capacities were obtained in the first discharging curve for the Zn-ferrite nanocorals; a decrease with the increase in current density was observed, as shown in Figure S6: 1809.1 F g^−1^ (2 A g^−1^), 1627.3 F g^−1^ (4 A g^−1^), 1540.9 F g^−1^ (6 A g^−1^), 1472.72 F g^−1^ (8 A g^−1^), and 1431.81 F g^−1^ (10 A g^−1^). The Coulombic efficiencies achieved were 95.4% (1 A g^−1^), 90.45% (2 A g^−1^), 90.40% (4 A g^−1^), 90.41% (6 A g^−1^), 95.3% (8 A g^−1^), and 96.92% (10 A g^−1^). Clearly, the Zn-ferrite nanocorals presented the best performance, prompting us to study it further.

**Figure 7:**
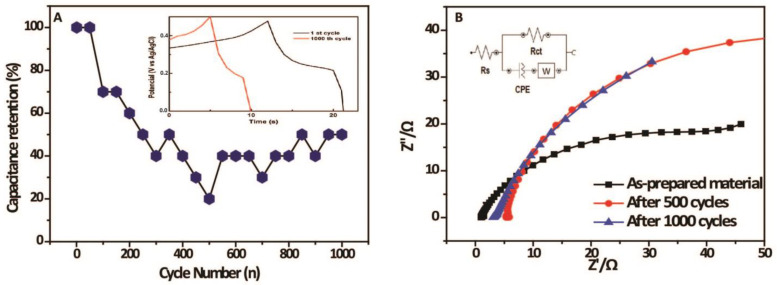
(**A**) Specific capacitances vs. number of cycles at 10 A g^−1^ (inset: GCD of the cycles 1 and 1000); (**B**) EIS analysis in the first, after 500 and 1000 charge–discharge cycling for the Zn-ferrite nanocorals (inset: equivalent electrical circuit).

Consequently, the Zn-ferrite nanocorals’ cycling stability was obtained by performing 1000 charge–discharge cycles with a current density of 10 A g^−1^. According to Figure 7A, we can note that the material had a considerable drop in its specific capacitance from the first to the four hundredth cycle. Then, the system kept its stabilization, presenting a specific capacitance of 268.8 F g^−1^ at the final cycle. Such phenomena could be explained due to irreversible redox reactions that may take place, damage, or loss of active sites. In addition, mechanical stress from electrolyte ions intercalation/deintercalation to counterbalance the overall charge can happen, affecting the efficiency of the material [50]. Initial charge–discharge cycles and the corresponding duration of charging and discharging at a current density of 1 A g^−1^ for the Zn-ferrite nanocorals are presented as an insert in Figure 7A. The charge–discharge curves for the first and thousandth cycles are not symmetrical. However, as the GCD curves in the following cycles demonstrate a high reversible charge–discharge course, we can affirm that the material presents excellent electrochemical capacitive behavior.

To understand this phenomenon further, EIS was performed at open circuit potential in 2.0 mol L^−1^ KOH electrolyte before, during (after 500 cycles), and after the 1000 cycles (Figure 7B). The charge transfer resistance obtained for the material before utilization was 68.4 Ω. It changed after 500 and 1000 cycles, presenting a value of 97.5 and 113.0 Ω, respectively, which explains the capacitance reduction after the 1000 cycles. Likewise, the plots’ slope changing suggests that there was some effect on the material’s surface [50].

Based on our findings, we can affirm that prepared porous Zn-ferrite nanocorals presented improved properties compared to ferrite materials in the literature. Table 1 shows the structure of the nanomaterials, synthesis process, electrolyte (and concentration), and specific capacitance at certain current densities. The morphology and synthesis method can undoubtedly affect a material’s performance. We could observe that the shape obtained in our experiments prompts remarkable results, achieving better performance than the examples presented in Table 1.

**Figure 8:**
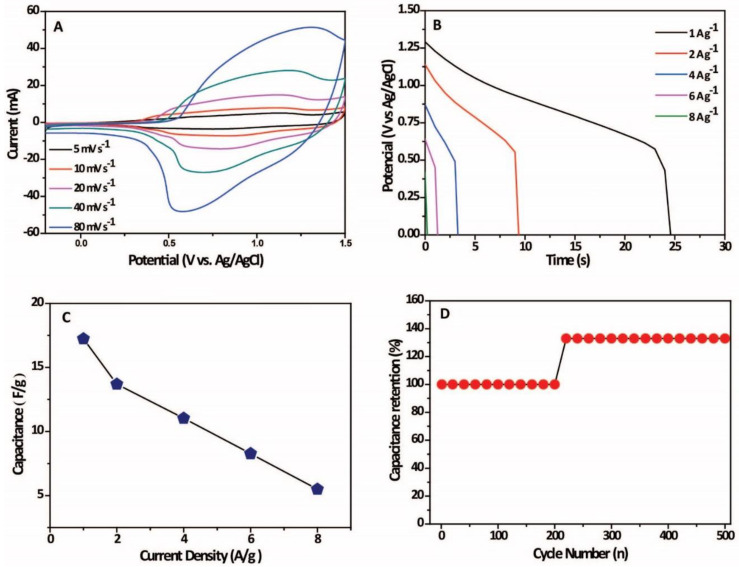
(**A**) The hybrid system’s CV curves at different scan rates (2.0 mol L^−1^ KOH electrolyte) and (**B**) Charge–discharge profiles at 1, 2, 4, 6, and 8 A g^−1^. (**C**) Specific capacitances vs. current densities. (**D**) Stability after 500 cycles at 6 A g^−1^.

Subsequently, we decided to evaluate the Zn-ferrite nanocorals’ performance on an Asymmetric Supercapacitor Cell (ASC). In this configuration, the prepared material was used as the anode, while the cathode was activated carbon. As a starting experiment, the operating voltage window of the ASC was assessed by CV (Figure 8A), in which the optimum voltage window was −0.2 to 1.5 V vs. Ag/AgCl, KClsat electrode. When the CV curves were performed at different scan ratios, a broadening of the redox peaks was observed as a battery-type Faradaic and capacitive electrode combination occurred. The CDC profiles raised the following results (Figure 8B): 17.2 F g^−1^ (1 A g^−1^), 13.7 F g^−1^ (2 A g^−1^), 11 F g^−1^ (4 A g^−1^), and 5.5 F g^−1^ (8 A g^−1^), with Coulombic efficiencies higher than 90%, which can be better followed by Figure 8C. The device offered a high energy density value of 18.1 Wh kg^−1^ at a power density of 2609.2 W kg^−1^ (at 1 A g^−1^ in 2.0 mol L^−1^ KOH electrolyte). We also performed 500 cycles, in which the ASC system showed a capacitance gain after 220 cycles, with stabilization up to the end of the cycles (Figure 8D).

## 3. Materials and Methods

Analytical-grade NaBr ≥ 99%, NaClO 12 wt%, TEMPO (2,2,6,6-tetramethyl-piperidinyl-*N*-oxy) 98%, ZnSO_4_·7H_2_O (≥99%), Fe_2_(SO_4_)_3_·7H_2_O (97%), and α-cellulose (99%) were purchased from Sigma-Aldrich and used without further purification. All solutions were prepared with deionized water (resistivity of 18.2 MΩ cm, Millipore^®^, Billerica, MA, USA). The morphology of the samples has been studied by Scanning electron microscopy (SEM) with JEOL JSM 7100F microscope (JEOL, Tokyo, Japan), operated at 15 kV; and by high-resolution transmission microscopy (HRTEM) with JEOL JEM 2100F microscope (JEOL, Tokyo, Japan), operated at 200 kV. Elemental mappings have been completed with energy-dispersive X-ray spectroscopy (EDS) for all the samples in STEM mode. The samples for microscopy studies were prepared by drop-casting isopropanol suspensions of the materials over silicon wafers (for SEM analysis) or carbon-coated copper grids (for HRTEM analysis), followed by drying under ambient conditions. Textural features were defined using nitrogen adsorption isotherms, recorded at −196 °C in a Micromeritics (Norcross, GA, USA) Gemini III 2375 surface area analyzer. Typically, 100 mg of each sample was degassed for 3 h at 150 °C prior to each analysis. We used the BET method to obtain the specific surface areas of the samples from the adsorption isotherms created in a relative pressure range of 0.07 < P/P_o_ < 0.3. The X-ray diffraction of the samples was obtained with an X’Pert PRO diffractometer (Philips, Amsterdam, The Netherlands), with an angle range between 10° and 95°, with a step of 0.02° during 6 s acquisition time. Fourier-Transform Infrared (FT-IR) spectra were attained using a Vertex 70 spectrometer V (Bruker, Billerica, MA, USA), with a resolution of 5 cm^−1^ in the region of 500–4000 cm^−1^. Raman measurements were carried out by a Horiba/Jobin-Yvon T64000 spectrometer (Horiba, Kyoto, Japan) equipped with a liquid N_2_-cooled CCD detector at a resolution of 2 cm^−1^ and excitation from a 532 nm wavelength solid-state laser (LAS-532-100-HREV) operating at 14 mW on the sample surface.

### 3.1. TCNF Synthesis

In a typical procedure for the synthesis of TCNF, 1 g of α-cellulose, 0.1 g of NaBr, 0.016 g of TEMPO (2,2,6,6-tetramethyl-piperidinyl-*N*-oxy), and 6.1 mL of 12 wt.% aqueous solution of NaClO were added to 100 mL of deionized water and were kept under stirring for 150 min. During the reaction, drops of 0.5 M NaOH were gradually added to maintain a pH between 10 and 11 [43]. Then, after 150 min, the aqueous suspension was centrifuged at 5800 rpm for 10 min, and the solid phase obtained was washed with deionized water. This procedure was repeated four times for each sample. Finally, the TCNF suspension was sonicated for 10 min to minimize agglomeration. The 15-mL suspension containing the separated solid was stored under refrigeration for use as a reactive template in the nano-oxide synthesis.

### 3.2. Synthesis of Nanocorals Mediated by TCNF

**Scheme 1:**
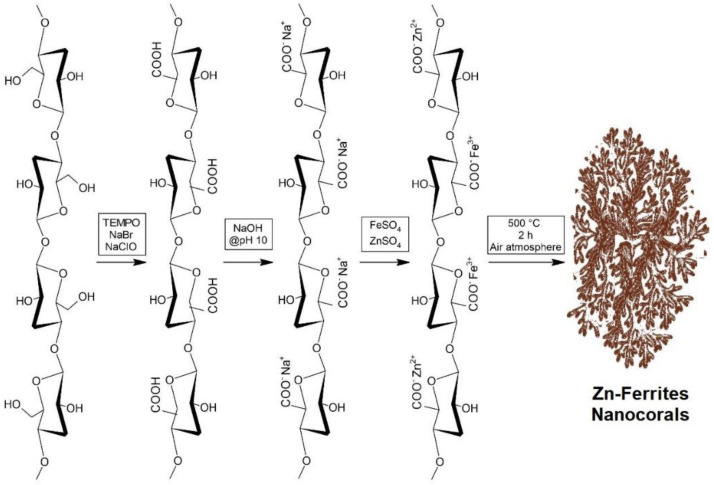
Illustrative synthetic route to produce Zn-ferrite nanocorals.

For synthesizing the nanostructured oxides, the previously obtained TCNF suspension was mixed under constant stirring with the desired metal-containing aqueous solution. In the case of the ZnO sample, an aqueous solution containing ZnSO_4_·7H_2_O in a concentration of 70 g L^−1^ was employed. For the Fe_2_O_3_ sample, Fe_2_(SO_4_)_3_·7H_2_O in a concentration of 90 g L^−1^ was employed. Finally, for the Fe_2_ZnO_4_ sample, an aqueous mixture containing both precursors in the same initial concentrations was used. After 60 min of contact time between 1 g of TCNF and each aqueous solution, the solid phase was vacuum filtered, and finally thermally treated under the presence of atmospheric air in a muffle furnace at 500 °C for 120 min, according to Scheme 1.

### 3.3. Electrode Preparation

The electrode preparation was started by cleaning the Nickel foam by immersing it in 3 mol L^−1^ HCl for 60 min, and subsequently washing it with isopropanol/water. Then, a suspension (also called ink, which is comprised of each material, performed separately) was made by mixing the sample, Super P, and polyvinylidene fluoride (PVDF) in N-methyl-2-pyrrolidone (NMP) at a weight ratio of 8:1:1. Following, 20 µL of each ink was transferred to the Nickel foam (previously cleaned) and dried overnight. After determining the mass of the active material of the foam (we weight the dried foam before and after the ink addition), Nickel wire was pressed on the edge of it, and the electrode was immersed in a 2.0 mol L^−1^ potassium hydroxide solution for 12 h previously to its utilization.

The specific capacitance (Cs, F g^−1^) of the studies was calculated according to Equation (1) [59]:

(1) $$\mathrm{Cs}=\frac{I\;\times \;\Delta t\;}{m\;\times \;\Delta v\;}$$

I (A) is related to the discharging current, ∆t (s) is the discharging time, m (g) represents the mass of material used for the working electrode obtaining, and ∆V (V) is the potential window.

The Coulombic efficiency (η) was calculated using Equation (2) [59]:

(2) $$\eta =\frac{t_{d}}{t_{c}}\;\times \;100\;\%$$

In the equation, t_d_ and t_c_ are the discharging and charging times, respectively.

#### Manufacture of Asymmetric Supercapacitor Cell

The Asymmetric Supercapacitor Cell (ASC) was assembled using Nickel foam loading Zn-ferrite nanocorals as the cathode and AC (Activated Charcoal) loading on Nickel foam as the anode; the electrolyte was KOH 2 mol L^−1^. The achieved Specific Capacitance from GCD analysis in a 3-electrode system was used to balance the charge/mass ratio between the cathode/anode. This charge/mass balance between the two electrodes follows the q+ = q− relationship to obtain the optimal performance system, where:

The charge stored (q) by each electrode depended on the following Equation (3) [59]:

(3) $$q=m\cdot C_{s}\cdot \Delta v$$

where C_s_ is specific capacitance, ∆V is the potential window of charge–discharge behavior, and m is the mass of the active electrode material. The active material weight loaded on the cathode for the ASC to obtain Q^+^ = Q^−^ was obtained using Equation (4) [60]:

(4) $$\frac{m_{+}}{m_{-}}=\frac{Q_{+}}{Q_{-}}$$

where m_+_ and m_−_ refer to the weights of the materials loaded on cathode and anode electrodes, respectively. Equations (5) and (6) were used to obtain the energy density (ED) and the power density (PD) [61]:

(5) $$\mathrm{ED}=\frac{1}{2}\cdot C_{s}{\left(\Delta V\right)}^{2}$$

(6) $$\mathrm{PD}=3600\;\times \;\frac{\mathrm{ED}}{\Delta t}$$

where C_s_ is the specific capacitance (F g^−1^) obtained in the GCD tests for the hybrid supercapacitor, ∆V (V) is the potential range between the cathode and anode, and ∆t (s) is the time of discharge of the device. All the tests were carried out at room temperature.

### 3.4. Electrochemical Measurements

The electrochemical measurements were performed using an Autolab PGSTAT 302 N potentiostat (Metrohm, Herisau, Switzerland) with NOVA 2.0 processing software. The working electrode comprised Nickel foam (0.5 mm thick, 1 cm × 2 cm) modified with the samples, in which a Nickel foil (0.125 mm thick, 1 mm × 3 mm) was pressed for conduction issues. The electrochemical measurements were performed in a three-electrode system using 2.0 mol L^−1^ potassium hydroxide media as an electrolyte, in which the working electrode (comprised of the prepared samples) was introduced into a single-compartment glass cell with Pt gauze as the auxiliary electrode and Ag/AgCl (KCl saturated) as the reference electrode. The Cyclic Voltammetry (CV) essays were recorded with the N_2_-saturated electrolyte, with different scan rates (5–80 mV s^−1^), in a potential range of −0.2 V to 0.5 V. The Galvanostatic Charge–discharge (GCD) tests were conducted under different current densities (1, 2, 4, 6, 8, and 10 A g^−1^). Electrochemical impedance spectroscopy (EIS) measurements were met by applying an AC voltage of 5 mV amplitude with a frequency range from 0.01 Hz to 100 kHz at open circuit potential.

## 4. Conclusions

A simple spinel Zn-ferrite nanocorals cellulose-mediate preparation was demonstrated. TEM, SEM, and HRTEM analyses of the Zn-ferrite nanocorals revealed the formation of multiple linked nanoparticles that grew as part of a spontaneous particle attachment process. Additionally, XRD and Raman studies showed that we could achieve a normal spinel structure, which probably was achieved due to the use of TCNF as physical templates for suitable adsorption of Zn^2+^ and Fe^3+^ cations. Such normal spinel structure could strongly affect the nanocorals’ performance, leading to superior supercapacitor performances relative to their monometallic counterparts, which was confirmed. The nitrogen adsorption–desorption isotherms of the material showed a high specific surface area and the obtaining of porous nanosystems, which is also highly desired for storage applications. Thus, a high specific capacity of 2031.81 F g^−1^ at a current density of 1 A g^−1^ was obtained, with good rate performance and excellent electrochemical stability (95.4%). We performed 1000 cycles, and even considering a decrease in the specific capacity, the material, after the hundredth cycle, kept stabilization up to the thousandth cycle.

We believe we have stepped forward to understand the utilization of biomass-derived spinel material for storage applications. As a personal opinion, although lithium cells have been widely used for power backup purposes, supercapacitors do not wear out or leak and can be recharged quickly. Even considering their lower storage ability compared to batteries, future studies are promising and aim to overcome such drawbacks. Bearing in mind our remarkable results, we intend to perform new experiments varying the metal precursors’ concentrations to achieve improved performances. Additionally, we intend to change Zn^2+^ ions for other ions of different metals, aiming to evaluate their effect on the storage process.

## Figures and Tables

**Table 1:** Comparison among the prepared material and some interesting examples in the literature.

Material	Structure	Synthesis	Electrolyte	Specific Capacitance (F g^−1^)	Current Density (A g^−1^)	Ref.
Zn-ferrite	Nanocorals	Thermal	2.0 M KOH	2031.8 F g^−1^	1.0 A g^−1^	This study
NiO@NiFe_2_O_4_	Nanoparticles	Hydrothermal	1.0 M KOH	248.4	1.0 A g^−1^	[51]
ZnFe_2_O_4_/RNG	Nanoparticles	Solvothermal	1.0 M KOH	244.0	0.5 A g^−1^	[48]
MnFe_2_O_4_/MoS_2_	Nanosheets	Hydrothermal	3.0 M KOH	2093.0	1.0 A g^−1^	[52]
MnZnFe_2_O_4_	Nanoneedles	Co-precipitation method	0.5 M H_2_SO_4_	550.0	0.8 A g^−1^	[15]
ZnFe_2_O_4_	Nanoflakes	Mechanochemical growth	3.0 M KOH	768.0	5.0 mA cm^−2^	[53]
ZnFe_2_O_4_ thin films	Nanospheres	Successive ionic layer adsorption and reaction method	6.0 M KOH	615.0	3 mA cm^−2^	[54]
ZnFe_2_O_4_	Nanosheets	Electrodeposition	6.0 M KOH	1093.0	1.0 A g^−1^	[55]
Ca_0.1_Zn_0.9_Fe_2_O_4_	Nanoparticles	Solvothermal	2.0 M Na_2_SO_4_	208.0	2.0 A g^−1^	[56]
ZnFe_2_O_4_	Nanofibers	Electrospun	3.0 M KOH	590.0	1.0 A g^−1^	[57]
ZnFe_2_O_4_	Microspheres	Solvothermal	1.0 M KOH	131.0	0.1 A g^−1^	[58]

## Data Availability

The authors confirm that the data supporting the findings of this study are available within the article [and/or] its Supplementary Materials.

## Supplementary Materials

The following supporting information can be downloaded at: https://www.mdpi.com/article/10.3390/ijms24119169/s1.
