# Supplementary material for: Sustainable Cellulose Nanofibers-Mediated Synthesis of Uniform Spinel Zn-Ferrites Nanocorals for High Performances in Supercapacitors
Source: Int J Mol Sci. 2023 May 24;24(11):9169. doi: 10.3390/ijms24119169 (PMC10252955; doi:10.3390/ijms24119169)
Supplement: Supplementary file 1 [file ijms-24-09169-s001.zip › ijms-2335760-supplementary.pdf]

International Journal of *Molecular Sciences*

MDPI

Support Information for

Sustainable Cellulose Nanofibers-Mediated Synthesis of Uniform Spinel Zn-ferrites Nanocorals for High Performances in Supercapacitors

Lucas T. Teixeira,[1] Scarllet L. S. de Lima,[1] Taissa F. Rosado,[1] Liying Liu,[2] Hector A. Vitorino,[3] Clenilton C. dos Santos,[4] Jhonatam P. Mendonça,[5] Marco A. S. Garcia,[5] Rogério N. C. Siqueira,[1] Anderson G. M. da Silva[1*].

[1] Departamento de Engenharia Química e de Materiais – DEQM, Pontifícia Universidade Católica do Rio de Janeiro, Rio de Janeiro/RJ, Brazil.
[2] Centro Brasileiro de Pesquisas Físicas, Rio de Janeiro/RJ, Brazil.
[3] Centro de Investigación en Biodiversidad para la Salud, Universidad Privada Norbert Wiener, Lima 15046, Peru.
[4] Departamento de Física, Centro de Ciências Exatas e Tecnologia, Universidade Federal do Maranhão, São Luís/MA, Brasil.
[5] Departamento de Química, Centro de Ciências Exatas e Tecnologia, Universidade Federal do Maranhão (UFMA), São Luís/ MA, Brazil.
* Correspondence: author: agms@puc-rio.br

Table S1. Textural properties measured by N_2-physisorption for the Zn-ferrite spinel, Fe_2O_3 and ZnO samples.

Sample	Specific Surface Area (m^2/g)	Total Pore Volume (cc/g)	Average Pore Diameter (nm)
Zn-ferrite spinel	33	0.19	24
Fe_2O_3	31	0.21	27
ZnO	20	0.08	17

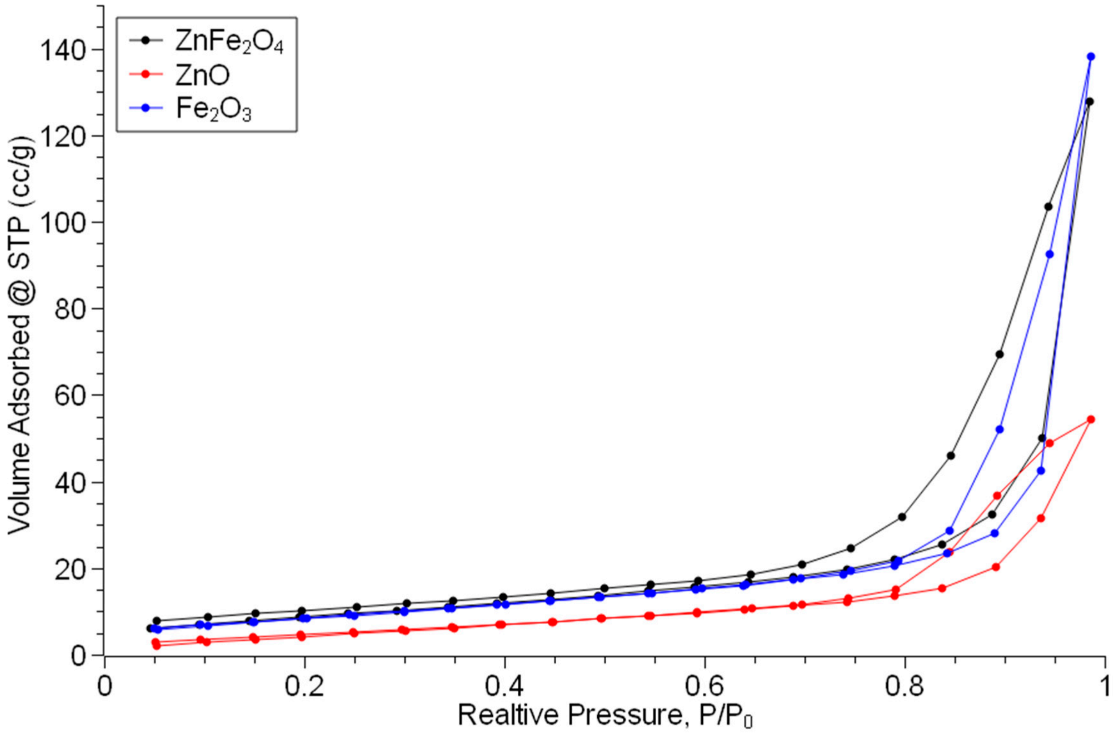

Figure S1. N_2 adsorption and desorption for Zn-ferrite ($ZnFe_2O_4$), Fe_2O_3, and ZnO nanocorals.

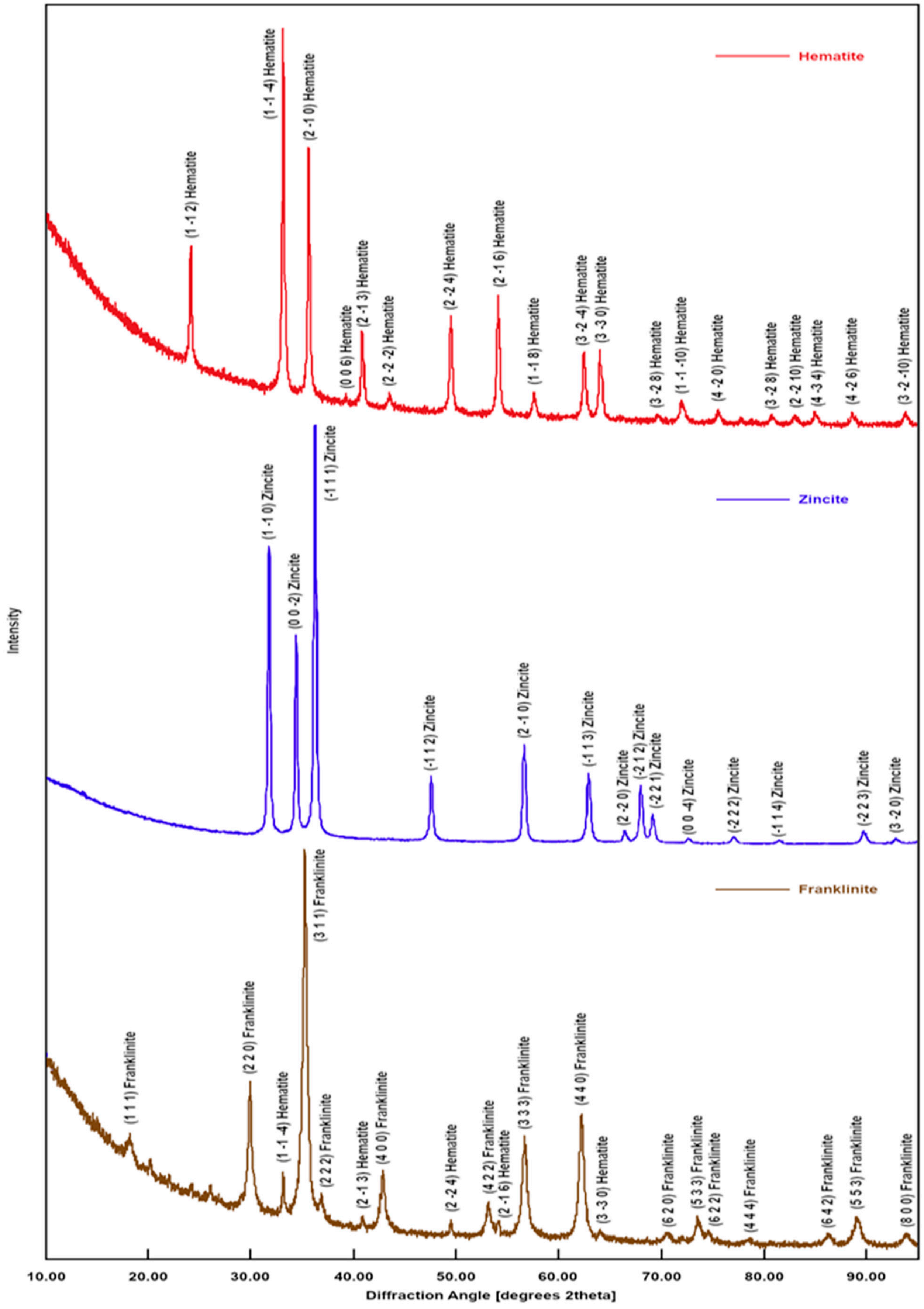

Figure S2. XRD and main diffraction peaks of Zn-ferrite, ZnO, and Fe_2O_3 samples.

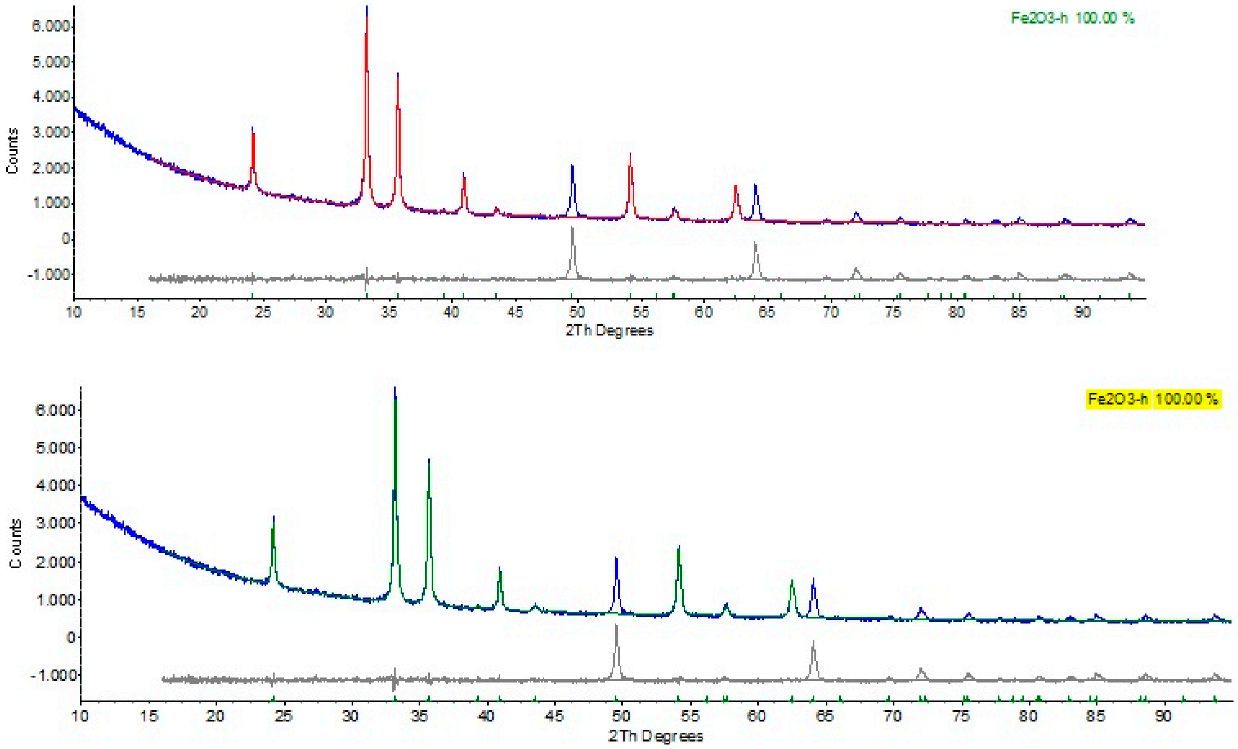

Figure S3. Rietveld refinement results for the Fe_2O_3 sample.

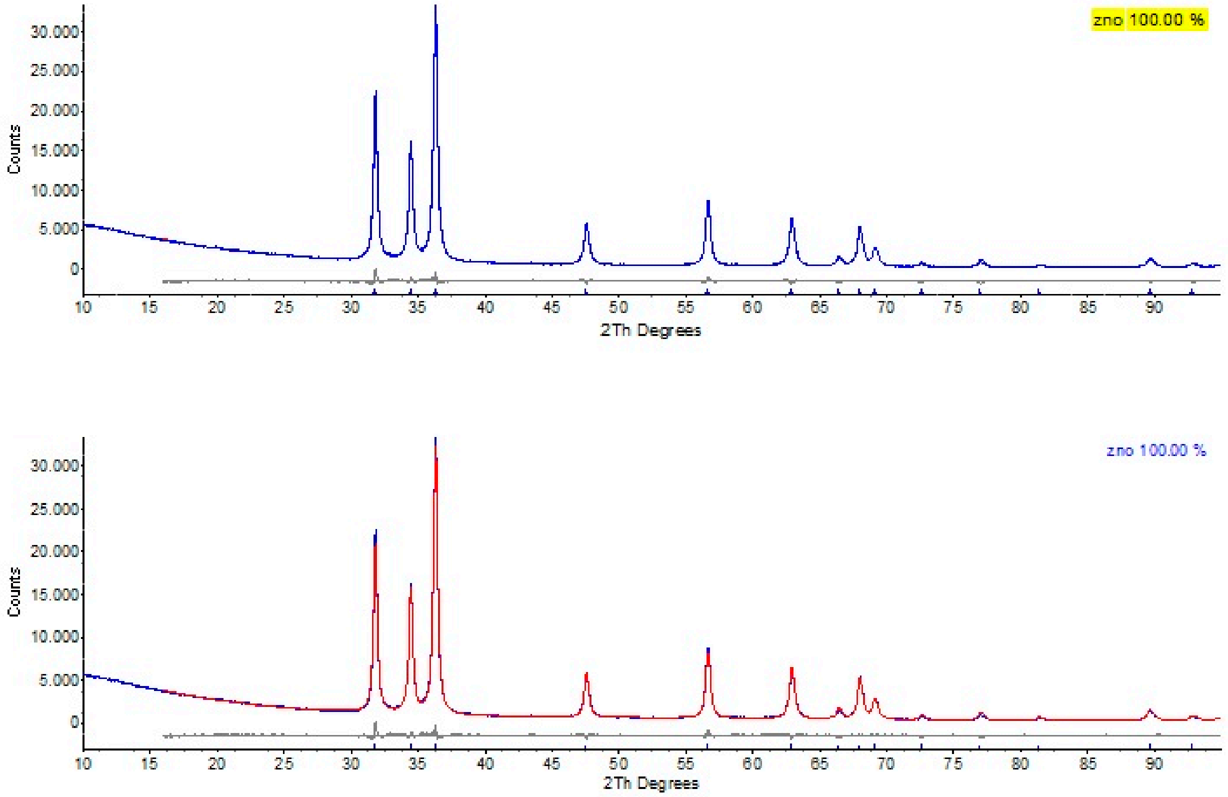

Figure S4. Rietveld refinement results for the ZnO sample.

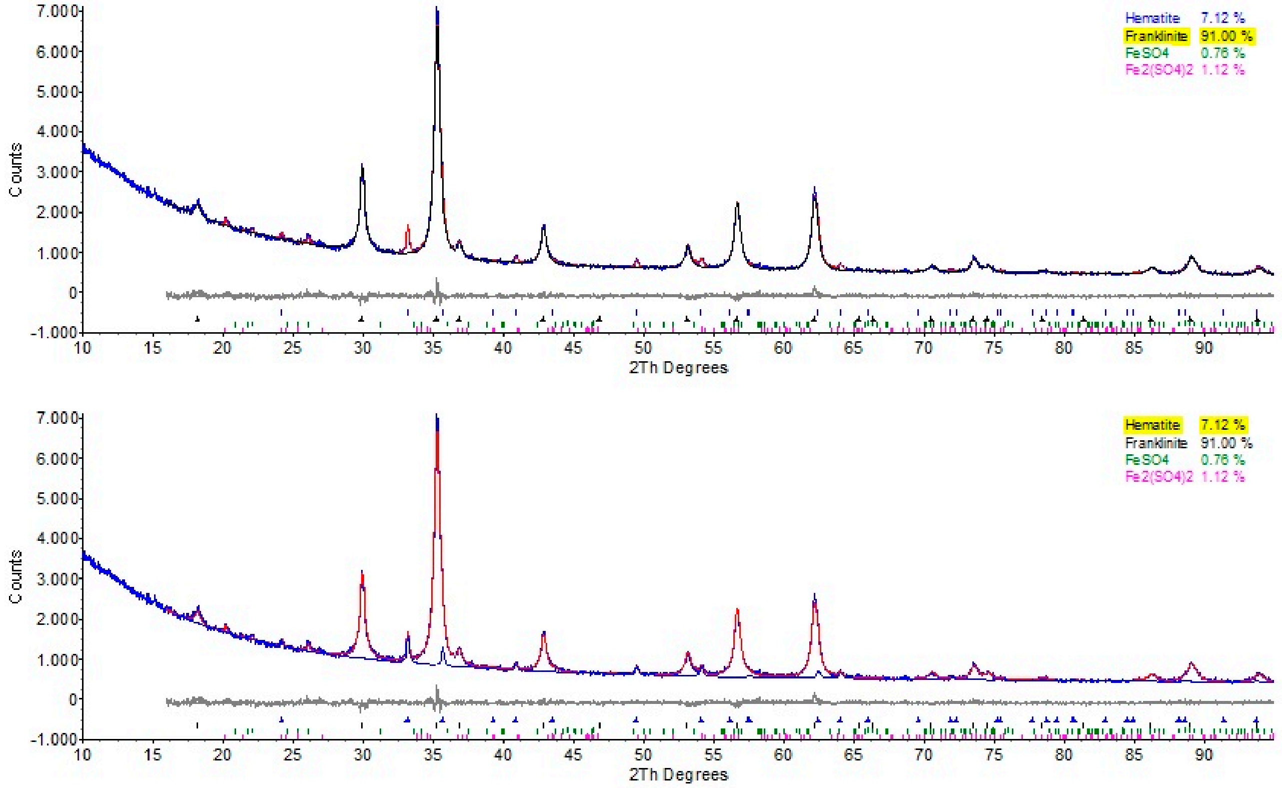

Figure S5. Rietveld refinement results for the Franklinite sample.

Table S2. Lattice parameters and mean crystallite sizes after Rietveld refinement for frakilinte, zincite and hematite crystalline phases.

Phase	Mean Crystallite Sizes (nm)	Refined Lattice Parameters (Angstron)	Literature Lattice Parameters (Angstron)
α-hematite (R3ch)	45.3	a=5.035; c=13.052	a=5.032; c=13.764 [49]
Zincite (P63mc)	26.3	a=3.250; c=5.207	a=3.252; c=5.212 [50]
Zn-ferrite (Fd3m)	52.8	a = 8.442	a=8.444 [51]

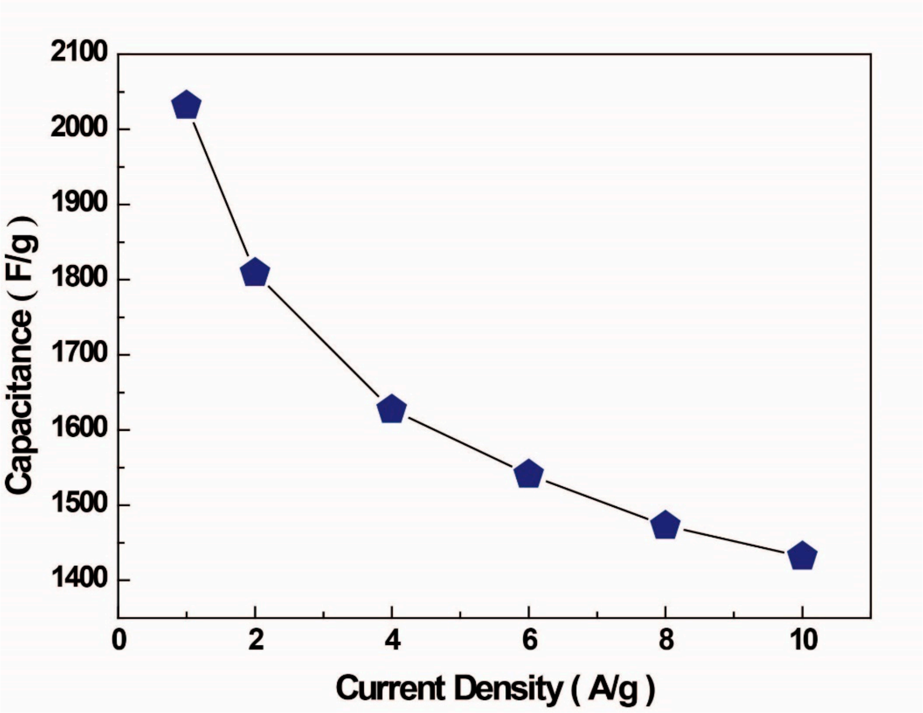

Figure S6. Capacitance vs. current density for the Zn-ferrite nanocorals.
